# Herd Health Program Participation Associated with Lower Vancomycin Resistance and Multidrug Resistance in Dairy Mastitis Pathogens: A Five-Year Surveillance Study in Saraburi, Thailand

**DOI:** 10.3390/biology15100782

**Published:** 2026-05-14

**Authors:** Sirirat Wataradee, Witaya Suriyasathaporn, Maneerat Somsee, Sukuma Samngamnim, Amonthep Khuprathumsiri, Kittisak Ajariyakhajorn, Thanasak Boonserm

**Affiliations:** 1 Department of Veterinary Medicine, Faculty of Veterinary Science, Chulalongkorn University, Bangkok 10330, Thailand; sirirat.w@chula.ac.th (S.W.);; 2 Dairy Research and Technology Transfer for Tropical Dairy Development Center (TDRC), Faculty of Veterinary Science, Chulalongkorn University, Saraburi 18110, Thailand; 3 Veterinary Academic Office, Faculty of Veterinary Medicine, Chiang Mai University, Chiang Mai 50200, Thailand; 4 Research Center of Producing and Development of Products and Innovations for Animal Health and Production, Chiang Mai University, Chiang Mai 50200, Thailand; 5 Overseas Campus, Asian Satellite Campuses Institute, Nagoya University, Nagoya 464-8601, Japan; 6 Large Animal Teaching Hospital, Faculty of Veterinary Science, Chulalongkorn University, Bangkok 10330, Thailand

**Keywords:** antimicrobial susceptibility, bovine mastitis, multidrug resistance, one health, rational drug use, Thailand

## Abstract

Antimicrobial resistance in bovine mastitis pathogens poses interconnected threats to dairy productivity and public health; however, longitudinal evidence on the association between veterinary oversight and lower resistance to critically important antimicrobials in tropical smallholder systems remains limited. This study analyzed 1347 quarter milk samples from 47 dairy farms in Saraburi Province, Thailand, submitted consecutively over five years (2020–2025), to evaluate whether participation in a veterinary-led herd health (HH) program was independently associated with reduced vancomycin resistance and multidrug resistance (MDR). Bacterial identification was conducted using standard methods, and susceptibility was determined using the Kirby–Bauer disk diffusion method. Among 1069 culture-positive samples, *Streptococcus* spp. (25.3%) and coagulase-negative staphylococci (16.8%) predominated. Vancomycin resistance declined from 15.9% to 5.4% between early (2020–2022) and recent (2023–2025) periods. Of the 686 isolates tested against ≥3 antibiotic classes, 61 (8.9%) were MDR; vancomycin resistance co-occurred in 34.4% of these samples. Repeated logistic regression identified non-HH farm status (OR = 4.035; *p* = 0.027) and early-year groups (OR = 4.611; *p* = 0.002) as independent risk factors for vancomycin resistance. As HH participation and year group showed a significant interaction, their joint effect for MDR was modeled: compared with non-HH farms in the early period (reference), all other combinations—including HH farms in 2023, HH farms in 2020, and non-HH farms in 2023—were associated with significantly lower odds of MDR. These findings provide the first evidence from Thailand that structured veterinary herd health programs are independently associated with lower critically important antimicrobial resistance, supporting their integration into a One-Health stewardship policy.

## Simple Summary

Bovine mastitis, which is routinely treated with antibiotics, is the costliest disease in dairy farming. When bacteria become resistant to these drugs, treatment fails and resistant organisms can spread to people through milk or direct contact. This study examined 1347 milk samples from 47 smallholder dairy farms in Saraburi Province, Thailand, over a five-year period (2020–2025). Some farms participated in a veterinary herd health program that included monthly veterinary visits, laboratory-based diagnosis before treatment, and farmer training on milking hygiene and responsible antibiotic use; other farms relied on symptom-based treatment without veterinary guidance. Farms outside the program were roughly four times more likely to harbor bacteria resistant to vancomycin—a last-resort antibiotic for serious human infections—and 2.5 times more likely to harbor multidrug-resistant bacteria. Resistance rates also fell markedly after 2023, when a regional Dairy School and local veterinary center were established. Yeast infections, a hallmark of antibiotic overuse, occurred only on non-program farms. These findings provide the first evidence from Thailand that structured veterinary oversight and farmer education are associated with lower rates of resistance to critically important antibiotics in dairy systems, supporting their adoption as part of national antimicrobial stewardship policy.

## 1. Introduction

Despite advances in husbandry practices, bovine mastitis remains the most economically significant infectious disease in dairy production, causing reduced milk yield, discarding of milk during treatment periods, premature culling, and increased veterinary costs [1,2]. Clinical mastitis reduces milk yield by an estimated 30–40% per lactation, while subclinical infections persistently elevate somatic cell counts (SCCs), compromising milk composition and quality [3]. Intramammary pathogens comprise contagious agents—principally *Staphylococcus aureus* and *Streptococcus agalactiae*—which are transmitted during milking, as well as environmental pathogens such as *Streptococcus uberis* and *Escherichia coli* [4]. Both groups increasingly harbor antimicrobial-resistant phenotypes, compounding treatment challenges and raising public health concerns, particularly where antibiotic use is inadequately regulated.

In Thailand, contagious pathogens—particularly *S. aureus* and *S. agalactiae*—persist as predominant mastitis isolates across multiple regions [5,6,7]. Antimicrobial therapy remains the primary treatment; however, escalating resistance increasingly undermines its efficacy [5]. Surveillance data across Southeast Asia have demonstrated progressive resistance to tetracyclines and beta-lactams in mastitis-associated bacteria, with resistance phenotypes exhibiting temporal trends and farm-level clustering consistent with selective pressure from localized antibiotic use [8,9]. Despite Saraburi Province’s significance as a major dairy hub in central Thailand, longitudinal data on pathogen distribution, resistance trends, and herd management factors in this region remain limited, thereby restricting the development of evidence-based antimicrobial stewardship.

Vancomycin resistance is of particular concern among Gram-positive pathogens, as the World Health Organization classifies vancomycin as a critically important antimicrobial (CIA). It is a last-resort agent for extensively resistant Gram-positive infections, including methicillin-resistant *S. aureus* (MRSA) and vancomycin-resistant enterococci (VRE) [10,11]. Although vancomycin has no therapeutic role in managing veterinary mastitis, phenotypic detection of non-susceptible isolates in livestock-associated bacteria carries implications for a One Health policy. This is because dairy systems serve as reservoirs for CIA resistance that is potentially transmissible to humans via food-chain or zoonotic pathways [12]. These phenotypic findings should be interpreted as surveillance signals rather than confirmed resistance mechanisms, pending molecular validation. Nonetheless, their relevance to public health is supported by prior reports: human cases of vancomycin-resistant *S. agalactiae* infection have been documented in the United States [13], and a bovine surveillance study from Thailand identified vancomycin-resistant *S. aureus* in milk samples [14].

This concern is compounded by the emergence of multidrug-resistant (MDR) phenotypes and increasing reports of mastitis-associated Gram-positive pathogens [14,15,16]. The co-occurrence of MDR with glycopeptide resistance, though uncommon, may be associated with organisms for which effective therapeutic options are severely limited should they enter human clinical settings [6,14]. These converging resistance trends underscore the need for systematic longitudinal surveillance in dairy systems where empirical antibiotic use remains prevalent.

Identifying modifiable management factors associated with AMR amplification or mitigation at the herd level is, therefore, a research priority. Evidence from small- and medium-sized dairy production contexts suggests that structured veterinary oversight and farmer education may be associated with reduced AMR burden when on-farm diagnostic infrastructure is limited [17,18]. Herd health (HH) programs—characterized by scheduled veterinary visits, diagnostic support, pathogen-specific treatment guidance, and farmer training on milking hygiene and rational drug use (RDU)—directly address the knowledge–practice gap sustaining empirical antibiotic overuse [19,20]. Veterinary oversight may support the appropriate selection of antibiotics by enabling a distinction between inflammatory and bacterial infections. Such knowledge can reduce unnecessary antibiotic use across a broad spectrum of contexts.

This study analyzed consecutive diagnostic submissions from dairy herds enrolled in a veterinary-led herd health program in Saraburi Province, Thailand, over a five-year surveillance period (2020–2025). The primary objective was to evaluate whether participation in a veterinary-led HH program was independently associated with reduced antimicrobial resistance and MDR among bovine mastitis pathogens. Secondary objectives included characterizing the distribution of pathogens, antimicrobial susceptibility profiles, and composition of MDR phenotypes across the surveillance period.

## 2. Materials and Methods

### 2.1. Study Setting and Farm Classification

This longitudinal study was conducted from January 2020 to May 2025, using data from 47 established dairy farms in Saraburi Province, central Thailand. All farms were within the service area of the general veterinary service of the Dairy Research and Technology Transfer for Tropical Dairy Development Center (TDRC), Faculty of Veterinary Science, Chulalongkorn University, Saraburi Province. The general veterinary service included regular training on dairy performance and improvements in raw milk quality, ambulatory veterinary services to provide emergency treatment, a disease diagnostic center, and a prototype demonstration for smallholder dairy farms. The farmers voluntarily participated in these services. In 2019, the TDRC established the Veterinary Service Innovation Project. It aimed to improve milk quality and control mastitis. Farmers could submit milk samples for bacterial identification and antimicrobial susceptibility testing. Under the project, some farmers voluntarily participated and enrolled in the Chulalongkorn University veterinary herd health program, in which project veterinarians visited the farm once a month to provide consultation on farm improvements. As a voluntary project, some farmers quit the herd health program but continued to use the TDRC service. Therefore, farms enrolled in the Chulalongkorn University (CU) veterinary herd health program with a cumulative participation duration of more than one year, signifying sufficient exposure to the herd health program, were designated HH farms. All remaining farms were designated non-HH farms. Sample classification was applied at the sample level; submissions from the same farm could therefore be classified differently depending on their cumulative duration at the time of submission. The classification criteria and the program components that distinguish HH from non-HH farms are detailed in Table 1.

Diagnostic submissions were further stratified by year group to evaluate temporal trends attributable to program expansion. Samples from 2020 to 2022 were assigned to the early period (Year 2020 group), corresponding to the program’s initial phase. Samples from 2023 to 2025 were assigned to the recent period (Year 2023 group), corresponding to an expanded phase following two key developments in 2023: (i) establishment of a regional Dairy School in Saraburi Province providing structured on-site farmer training in mastitis management and rational drug use, and (ii) establishment of the TDRC satellite center within Saraburi Province, enabling direct local veterinary oversight.

**Table 1:** Program components and management characteristics distinguishing HH farms from non-HH farms in the Veterinary Service Innovation Project, Saraburi Province, Thailand (2020–2025). HH farms were enrolled in the structured herd health program with a cumulative participation of ≥1 year; non-HH farms were either not enrolled or participated for <1 year.

Characteristic	HH Farm	Non-HH Farm
**Farm classification**		
Number of farms	15	32
Classification criteria ^a^	CU-enrolled; HH participation >1 year	CU-enrolled <1 year or not enrolled
Diagnostic submissions	325	1022
**Program components**		
Veterinary visits	Scheduled monthly (≥1 visit/month)	As-needed only
Bulk tank milk quality monitoring	Monthly: Somatic cell count (SCC), Total Plate Count, Lab Pasteurized Count, and coliform count	None
Individual cow monitoring	Monthly: milk composition and SCC	None
Pathogen identification	Bacteriological culture when SCC was elevated or CM was detected	None; symptom-based only
Antibiotic use policy	RDU—culture-guided, targeted selection	Empirical—unguided
Management approach	Preventive and proactive	Reactive
Milking protocol	Standardized and regularly reviewed	Inconsistent
Post-milking teat dipping	Systematic protocol; monitored	Irregular or absent
Farmer education	Formal training: milking hygiene, mastitis recognition, and RDU	None
Record keeping	Individual cow health records	Minimal or absent

^a^ The program duration is calculated as the interval between the first and last diagnostic submission dates for each farm. Sample classification is applied at the sample level: submissions from CU-enrolled farms with cumulative duration from first submission <1 year were classified as a non-HH farm for that submission, reflecting the period before sufficient program exposure. Subsequent submissions from the same farm at a cumulative duration >1 year were classified as a HH farm.

### 2.2. Sample Collection and Handling

Quarter milk samples were aseptically collected by attending veterinarians or trained farm personnel from cows with clinical mastitis (CM), defined as visible abnormalities in the milk, udder, systemic signs, or subclinical mastitis (SCM), defined as a California Mastitis Test score > 1. All samples were kept on ice and transported to the Mastitis and Milk Quality Laboratory at the Livestock Hospital, Faculty of Veterinary Science, Chulalongkorn University, where bacterial identification was performed within 24 h. Samples with positive bacterial culture results were then subjected to antimicrobial susceptibility testing. For each isolate, approximately 3–5 agents were selected by the attending veterinarian based on bacterial species, clinical presentation, and herd treatment history, consistent with routine diagnostic practice in resource-limited settings [16].

### 2.3. Bacterial Isolation and Identification

The collected milk samples were cultured on Columbia blood agar supplemented with 5% bovine blood and MacConkey agar and incubated aerobically at 37 °C for 24–48 h. Identification followed a standardized three-tier protocol: (i) colony morphology; (ii) Gram staining; and (iii) biochemical characterization, including catalase, oxidase, the CAMP test, and carbohydrate fermentation assays, according to the NMC Laboratory Handbook on Bovine Mastitis [21]. Isolates of *Streptococcus* spp. were further differentiated by esculin hydrolysis; esculin-positive isolates were provisionally classified as *Streptococcus* spp. (esculin+) pending API confirmation, while esculin-negative streptococci were identified as *S. agalactiae* or *S. dysgalactiae* by the CAMP test. *Corynebacterium bovis* and Gram-negative organisms other than *E. coli* were classified as “Other”. Samples yielding no growth were recorded as culture-negative, and those with ≥3 morphologically distinct colony types alongside environmental *Bacillus* spp. were recorded as contaminated.

### 2.4. Antimicrobial Susceptibility Testing (AST)

Antimicrobial susceptibility was determined using the Kirby–Bauer disk diffusion method following CLSI VET01 guidelines [22]. The panel comprised agents representing eight pharmacological classes: penicillin (ampicillin 10 µg; penicillin G 10 µg), fluoroquinolones (enrofloxacin 5 µg), aminoglycosides (gentamicin 10 µg), potentiated sulfonamides (sulfamethoxazole–trimethoprim 23.75/1.25 µg), tetracyclines (tetracycline 30 µg), cephalosporins (cefoxitin 30 µg; cefotaxime 30 µg; ceftiofur 30 µg; ceftriaxone 30 µg), macrolides (erythromycin 15 µg), and glycopeptides (vancomycin 30 µg). Vancomycin was included solely as a CIA surveillance marker; it has no therapeutic role in veterinary mastitis management and is not recommended by CLSI as a routine diagnostic test for livestock isolates. Therefore, the results are interpreted as indicative of phenotypic non-susceptibility only and do not constitute evidence of clinically relevant or transferable resistance without supporting MIC data and molecular characterization of resistance determinants.

Inhibition zone diameters were measured after 24 h at 37 °C and interpreted as susceptible (S), intermediate (I), or resistant (R) as per the CLSI breakpoint. Intermediate isolates were combined with susceptible (S + I) as the reference category, with resistant (R) as the outcome.

### 2.5. Statistical Analysis

Data are reported as frequencies, percentages, and odds ratios where appropriate. Data were collected and organized by Microsoft Excel (Microsoft Corporation, Redmond, WA, USA), and statistical analyses were performed using SAS OnDemand for Academic (ver. 9.4) (SAS Institute Inc., Cary, NC, USA). The associations of mastitis occurrence stratified by bacterial pathogens, based on the total number of collected samples, with independent variables, including mastitis type (CM vs. SCM), HH status (HH farm vs. non-HH farm), and starting participation year group (2020 vs. 2023), were determined using the chi-square test or Fisher’s exact test, as appropriate. Based on the number of samples with positive bacterial identification after using a specified number of antimicrobial agents, the association between resistant percentages of specified antimicrobial agents, independent variables, and bacterial pathogens was evaluated using Fisher’s exact chi-squared test.

Multidrug-resistant bacteria (MDR) are defined as bacteria that are immune to at least one agent in three or more antimicrobial classes [15]. Due to antibiotic agent selection by the veterinarians, bacteria tested with fewer than 3 antibiotic classes were excluded from the final analysis. The MDR phenotype screening indicators were determined using sensitivity, specificity, positive predictive value (PPV), negative predictive value (NPV), and overall accuracy. Due to the use of multiple samples from the same farms, factors associated with MDR were determined using multiple repeated logistic regression (Proc genmod, SAS Academic Software (SAS Institute Inc., Cary, NC, USA)) with the free-entering method. Samples nested within the same farm were treated as a repeated factor, and an exchangeable correlation structure was used, since all cows in the same farm were managed similarly. The free-entering method was used to control any confounding factors in the model. Because some farms were involved in establishing the TDRC satellite center at different times, the interaction between the HH farm and the participating year was evaluated for each model. The interaction effect, rather than the separate factors, was included in the model due to its significance. Factors with *p* < 0.1 were used and retained in the model. Statistical significance was set at *p* < 0.05.

## 3. Results

### 3.1. Sample Distribution and Pathogen Profile

**Figure 1:**
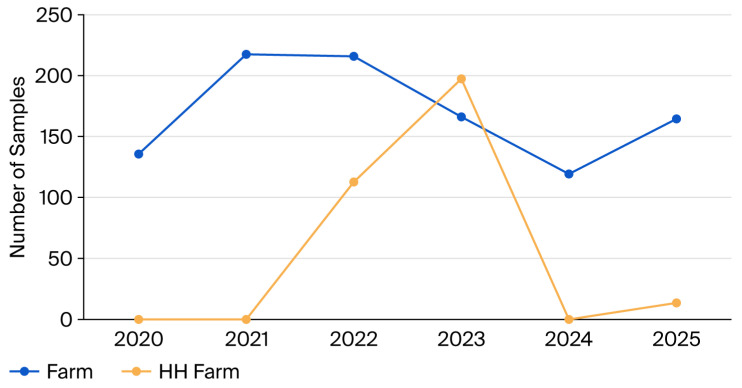
The number of milk samples collected during the study was stratified by farm: farms without participation in a herd health program (non-HH farm) and farms with HH participation for more than 1 year (HH farm).

A total of 1347 quarter milk samples from 47 dairy farms were analyzed across 2020–2025, peaking in 2023 (*n* = 365). Samples classified as belonging to a HH farm at submission totaled 325 (24.1%), with the proportion increasing from 2021 onward following the expansion of the program and establishment of the TDRC regional center (Figure 1). Due to insufficient data, samples classified as “other” types of mastitis (*n* = 9) were excluded.

From all the submissions, 1069 (79.4%) yielded bacterial growth, 275 (20.4%) were culture-negative, and 3 (0.2%) were contaminated. The predominant isolates were *Streptococcus* spp. (*n* = 341, 25.3%), coagulase-negative staphylococci (CNS; *n* = 226, 16.8%), and *S. uberis* (*n* = 106, 7.9%). Contagious pathogens comprised *S. agalactiae* (*n* = 74, 5.5%) and *S. aureus* (*n* = 16, 1.2%), while *S. dysgalactiae* (*n* = 88, 6.5%) was identified as an additional streptococcal isolate. Yeast accounted for 3.0% of submissions (*n* = 41; Table 2).

Statistical analysis revealed significant associations between pathogen distribution and all three factors. *S. aureus* (OR = 6.0, *p* < 0.01), *S. uberis* (OR = 2.7, *p* < 0.01), and *E. coli* (OR = 5.0, *p* < 0.01) were disproportionately associated with CM, while *S. agalactiae* predominated in cases of SCM (OR = 0.2, *p* < 0.01). Regarding HH participation, *S. uberis* was markedly more prevalent in HH farm submissions (OR = 10.6, *p* < 0.01), while *S. dysgalactiae* was less frequently detected (OR = 0.5, *p* < 0.01). Yeast was exclusively isolated from non-HH farm submissions (*p* < 0.01). Temporally, *S. agalactiae* (OR = 7.2, *p* < 0.01) and *S. uberis* (OR = 2.8, *p* < 0.01) increased in the 2023 year group, while *S. dysgalactiae* declined (OR = 0.3, *p* < 0.01).

**Table 2:** Association between mastitis pathogens and types of mastitis (clinical and subclinical) by years of participation (2020–2022 (2020), 2023–2025 (2023)) in a herd health program (without HH (farm), with HH (HH farm)). ***, **, * indicate significant associations at *p* < 0.01, <0.05, and <0.1, respectively.

	Total	CNS	*S. aureus*	*S. agalactiae*	*S. dysgalactiae*	*S. uberis*	*E. coli*	Streptococci	Other	Yeast	Overall
		*n* (%)	*n* (%)	*n* (%)	*n* (%)	*n* (%)	*n* (%)	*n* (%)	*n* (%)	*n* (%)	*n* (%)
n		226	16	74	88	106	36	341	141	41	1347
Mastitis type											
SCM	983	176 (17.9)	6 (0.6)	68 (6.9)	61 (6.2)	55 (5.6)	13 (1.3)	254 (25.8)	118 (12.0)	37 (3.8)	788 (80.2)
CM	364	50 (13.7)	13 (3.6)	6 (1.7)	27 (7.4)	51 (14.0)	23 (6.3)	87 (23.9)	23 (6.3)	4 (1.1)	284 (78.0)
OR		0.7 *	6.0 ***	0.2 ***	1.2	2.7 ***	5.0 ***	0.9	0.5 ***	0.3 **	0.9
Participation in the herd health program											
Non-HH Farm	1022	169 (16.5)	9 (0.9)	42 (4.1)	75 (7.3)	29 (2.8)	25 (2.5)	290 (28.4)	120 (11.7)	41 (4.0)	800 (78.3)
HH Farm	325	57 (17.5)	10 (3.1)	32 (9.9)	13 (4.0)	77 (23.7)	11 (3.4)	51 (15.7)	21 (6.5)	0 (0)	272 (83.7)
OR		1.1	3.6 **	2.5 ***	0.5 **	10.6 ***	1.4	0.5	0.5 ***	N.A. ***	1.4 **
Year of participation											
2020	683	115 (16.8)	5 (0.7)	10 (1.5)	67 (9.8)	30 (4.3)	22 (3.2)	204 (30.0)	77 (11.3)	19 (2.8)	549 (80.4)
2023	664	111 (16.7)	14 (2.1)	64 (9.6)	21 (3.2)	76 (11.5)	14 (2.1)	137 (20.6)	64 (9.6)	22 (3.3)	523 (78.8)
OR		1	2.9 **	7.2 ***	0.3 ***	2.8 ***	0.6	0.6 ***	0.8	1.2	0.9

### 3.2. Antimicrobial Resistance Profiles

A further limitation concerns the heterogeneity of the AST panel. Because antibiotic agents were selected per isolate by the attending veterinarian (3–5 agents), the number of represented classes varied across isolates. Isolates tested against fewer than three antibiotic classes were excluded from MDR classification (*n*  =  686 eligible samples of 1069 culture-positive samples), likely underestimating true MDR prevalence. Agent-level resistance rates in Table 3 reflect variable denominators and should only be compared across groups with reference to the sample sizes shown. For MDR classification and regression analyses (Table 4, Table 5, Table 6 and Table 7), only isolates tested against ≥3 antibiotic classes simultaneously were included (*n* = 686), ensuring sufficient data for the enumeration of resistance classes per the applied MDR definition.

Among isolates tested against ≥3 antibiotic classes, sulfonamides and tetracyclines recorded the highest resistance frequencies at 39.7% (48/121) and 36.8% (163/443), respectively, followed by cefotaxime (24.0%; 123/513), erythromycin (20.9%; 83/397), gentamicin (14.3%; 47/328), enrofloxacin (14.6%; 30/206), vancomycin (12.4%; 42/338), and penicillins (ampicillin 11.4%, 74/648; penicillin G 10.6%, 43/405). Agents tested against <100 isolates should be interpreted cautiously (Table 3).

Among individual antibiotic classes, *S. uberis* exhibited significantly elevated aminoglycoside resistance (gentamicin; OR = 7.6, *p* < 0.01) and tetracycline resistance (OR = 4.2, *p* < 0.01), and carried the highest within-species MDR rate among all pathogens (11.5%; see Section 3.3). However, this descriptive resistance burden does not translate directly to an independent risk in the adjusted regression model, where *S. uberis* was associated with lower MDR odds after controlling for HH × year effects (Table 7); this apparent inversion is examined. *S. agalactiae* was uniformly susceptible to penicillins (OR = N.A., *p* < 0.01) with lower cephalosporin resistance (OR = 0.3, *p* < 0.01). Staphylococci exhibited high penicillin resistance (CNS: OR = 16.8; *S. aureus*: OR = 15.7; both *p* < 0.01). Vancomycin resistance frequencies and odds ratios were calculated for Gram-positive isolates only. The elevated OR for *E. coli* (*n*  =  10) reflects intrinsic outer-membrane impermeability to glycopeptides; *E. coli* was excluded from vancomycin resistance calculations and regression analyses.

**Figure 2:**
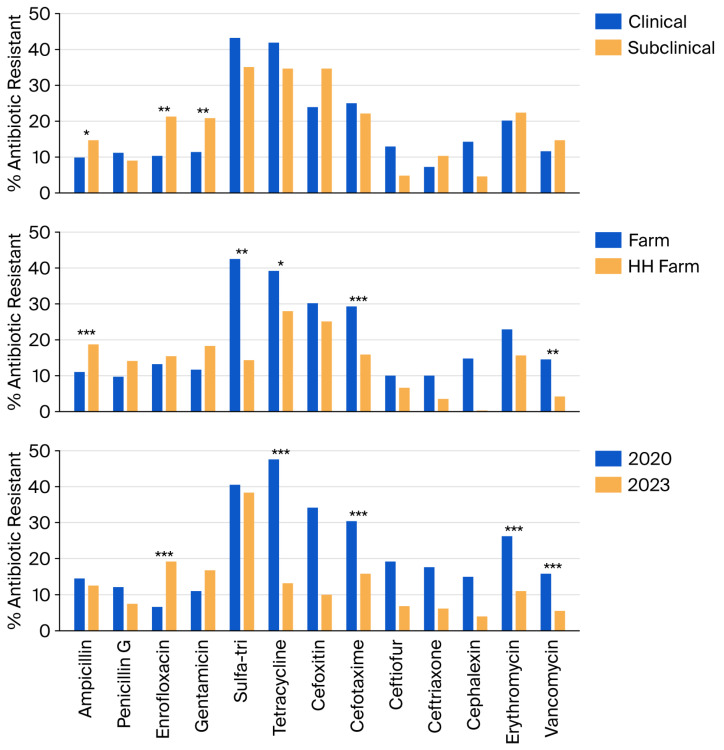
Association between percentages of antibiotic resistance in mastitis pathogens with mastitis types (clinical, subclinical), participation in a herd health program (without HH participation (non-HH farm), with HH participation (HH farm)), and year of participation (2020–2022 (2020), 2023–2025 (2023)). ***, **, * indicated significant association at *p* < 0.01, <0.05, and <0.1, respectively.

Stratified analysis demonstrated that year group was the most consistent source of variation in resistance (Figure 2). Resistance against tetracycline, erythromycin, cephalosporin, and vancomycin declined significantly from 2020 to 2023 (all *p* < 0.01). Enrofloxacin resistance increased across the same interval (*p* < 0.05). Erythromycin resistance was significantly lower in isolates from HH farms than from non-HH farms (*p* < 0.01), while tetracycline resistance was paradoxically higher (*p* < 0.05). Vancomycin resistance did not differ significantly between isolates from non-HH farms and HH farms at the bivariate level.

### 3.3. MDR Phenotype Characterization

Of the 686 isolates included in the AST subset, 61 (8.9%) met the MDR criteria. Three-class resistance predominated (*n* = 48), with TET-CEP-ERT identified as the most frequent pattern (*n* = 14), followed by SFT-CEP-VAN (*n* = 9) and CEP-ERT-VAN (*n* = 4). Four-class MDR was found to comprise 10 isolates, and five-class MDR comprised four isolates, three of which exhibited the PEN-TET-CEP-ERT-VAN pattern (Table 4).

Of the 61 MDR isolates included, 21 (34.4%) harbored concurrent vancomycin resistance across all resistance complexity levels. *Streptococcus* spp. collectively accounted for the largest proportion of MDR (52.5%); descriptively, *S. uberis* exhibited the highest within-species MDR rate (11.5%), although *S. agalactiae* contributed no MDR isolates (0%).

**Table 3:** Number (percentage) of antibiotic susceptibility tests conducted against bacteria causing bovine mastitis in milk samples. ***, **, * indicate association levels at *p* < 0.01, <0.05, and <0.1, respectively.

		Total	CNS	*S. aureus*	*S. agalactiae*	*S. dysgalactiae*	*S. uberis*	*E. coli*	Streptococci	Other
Ampicillin	S-I	574	34 (5.9)	7 (1.2)	65 (11.3)	65 (11.3)	85 (14.8)	14 (2.4)	192 (53.0)	17 (4.7)
	R	74	38 (51.4)	12 (16.2)	0 (0)	0 (0)	2 (2.7)	11 (14.9)	9 (20.9)	4 (9.3)
	OR		16.8 ***	15.7 ***	N.A. ***	N.A. ***	0.16 ***	7.0 ***	0.2 ***	2.1
Penicillin	S-I	362	22 (6.1)	2 (0.6)	32 (8.8)	80 (13.9)	40 (11.1)	0 (0)	279 (49.5)	3 (0.5)
	R	43	25 (58.1)	3 (7.0)	0 (0)	0 (0)	1 (2.3)	0 (0)	14 (15.9)	7 (8.0)
	OR		21.5 ***	13.5 ***	N.A. **	N.A. ***	0.2	N.A.	0.2 ***	16.1 ***
Enrofloxacin	S-I	176	46 (26.1)	3 (1.7)	33 (18.8)	9 (5.1)	39 (22.2)	12 (6.8)	29 (16.5)	5 (2.8)
	R	30	11 (36.7)	4 (13.3)	0 (0)	2 (6.7)	3 (10.0)	1 (3.3)	9 (30.0)	0 (0)
	OR		1.6	8.9 ***	N.A. ***	1.3	0.4	0.5	2.2	N.A.
Gentamicin	S-I	281	60 (21.4)	7 (2.5)	48 (17.1)	12 (4.3)	25 (8.9)	18 (6.4)	72 (26.6)	28 (10.4)
	R	47	5 (10.6)	0 (0)	0 (0)	0 (0)	20 (42.6)	1 (2.1)	19 (41.3)	1 (2.2)
	OR		0.4	N.A.	N.A. ***	N.A. ***	7.6 ***	0.3	1.9 *	1.9 *
Sulfa-tri	S-I	73	5 (6.9)	6 (8.2)	0 (0)	9 (12.3)	0 (0)	4 (5.5)	36 (49.3)	13 (17.8)
	R	48	2 (4.2)	0 (0)	0 (0)	4 (8.3)	1 (2.1)	0 (0)	31 (66.0)	9 (19.2)
	OR		0.6	N.A. *	N.A.	0.6	N.A.	N.A.	1.9 *	1.1
Tetracycline	S-I	280	34 (12.1)	5 (1.8)	17 (6.0)	38 (13.6)	12 (4.3)	6 (2.1)	157 (56.3)	10 (3.6)
	R	163	6 (3.7)	0 (0)	0 (0)	26 (16.0)	26 (16.0)	3 (1.8)	89 (54.6)	13 (8.0)
	OR		0.3 ***	N.A.	N.A. ***	1.2	4.2 ***	0.86	0.9	2.3 *
Cefoxitin	S-I	36	2 (5.6)	11 (30.6)	0 (0)	3 (8.3)	1 (2.8)	4 (11.1)	10 (27.8)	5 (13.9)
	R	15	3 (20.0)	0 (0)	0 (0)	1 (6.7)	0 (0)	5 (33.3)	3 (20.0)	3 (20.0)
	OR		4.3	N.A. **	N.A.	0.8	N.A.	4	0.65	1.6
Cefotaxime	S-I	390	62 (15.9)	15 (3.9)	51 (13.1)	63 (16.1)	46 (11.8)	17 (4.4)	116 (29.7)	20 (5.1)
	R	123	1 (0.8)	0 (0)	5 (4.1)	5 (4.1)	31 (25.2)	0 (0)	75 (61.0)	6 (4.9)
	OR		0.04 ***	N.A. **	0.3 ***	0.2 ***	2.5 ***	N.A. **	3.7 ***	0.9
Ceftiofur	S-I	87	7 (8.1)	15 (17.2)	8 (9.2)	5 (5.8)	9 (10.3)	10 (11.5)	30 (34.9)	2 (2.3)
	R	9	1 (11.1)	0 (0)	0 (0)	0 (0)	0 (0)	3 (33.3)	5 (55.6)	0 (0)
	OR		1.4	N.A.	N.A.	N.A.	N.A.	3.9	2.3	N.A.
Ceftriaxone	S-I	91	21 (23.1)	0 (0)	0 (0)	5 (5.5)	2 (2.2)	7 (7.7)	50 (55.0)	6 (6.6)
	R	8	0 (0)	0 (0)	0 (0)	3 (37.5)	1 (12.5)	0 (0)	3 (37.5)	1 (12.5)
	OR		N.A.	N.A.	N.A.	10.3 **	6.4	N.A.	0.5	2
Cephalexin	S-I	64	12 (18.8)	1 (1.6)	0 (0)	6 (9.4)	3 (4.7)	4 (6.3)	21 (32.8)	17 (26.6)
	R	8	1 (12.5)	2 (25.0)	0 (0)	0 (0)	0 (0)	0 (0)	1 (12.5)	4 (50.0)
	OR		0.6	21.0 **	N.A.	N.A.	N.A.	N.A.	0.3	2.8
Erythromycin	S-I	314	12 (3.8)	4 (1.3)	13 (4.1)	60 (19.1)	37 (11.8)	7 (2.2)	157 (52.2)	11 (3.7)
	R	83	2 (2.4)	3 (3.6)	0 (0)	12 (14.5)	8 (9.6)	4 (4.8)	50 (61.7)	2 (2.5)
	OR		0.6	2.9	N.A. *	0.7	0.8	2.2	1.5	0.7
Vancomycin	S-I	296	2 (0.7)	1 (0.3)	29 (9.8)	51 (17.2)	34 (11.5)	6 (2.0)	172 (58.1)	1 (0.3)
	R	42	1 (2.4)	0 (0)	3 (7.1)	6 (14.3)	5 (11.9)	4 (9.5)	23 (54.8)	0 (0)
	OR		3.6	N.A.	0.7	0.8	1	N.A.	0.9	N.A.

### 3.4. Multidrug Resistance, Screening Indicators, and Associated Factors

Individual resistance phenotypes were evaluated as exploratory MDR screening indicators (Table 5). Vancomycin resistance demonstrated the highest specificity (93.3%) and PPV (52.4%), while sulfonamide and tetracycline resistance provided the highest sensitivity (90.9% and 88.9%, respectively). Overall accuracy was highest for enrofloxacin (89.3%), vancomycin (89.1%), and cephalosporins (84.7%).

For vancomycin resistance (*n* = 338; Table 6), non-HH farms (OR = 4.035; 95% CI: 1.175–13.860; *p* = 0.027) and the 2020 year group (OR = 4.611; 95% CI: 1.709–12.441; *p* = 0.002) were independently associated with higher odds of resistance. For MDR (*n* = 686; Table 7), *S. uberis* was associated with lower adjusted odds of MDR (OR = 0.246; 95% CI: 0.070–0.863; *p* = 0.029), and *S. dysgalactiae* was associated with higher adjusted odds of MDR (OR = 2.792; 95% CI: 1.136–6.861; *p* = 0.025). Because the interaction between HH participation and year group was significant, it replaced the separate main effects in the final model. Compared with non-HH farms in the early period (reference), the three other groups were associated with significantly lower odds of MDR: HH farms in 2023 (OR = 0.135; 95% CI: 0.053–0.348; *p* < 0.0001), HH farms in 2020 (OR = 0.065; 95% CI: 0.021–0.206; *p* < 0.0001), and non-HH farms in 2023 (OR = 0.184; 95% CI: 0.064–0.531; *p* = 0.002).

**Table 4:** Multidrug-resistant (MDR) patterns of bacteria from bovine milk samples (*n* = 61), including penicillin groups (PEN), summarized for ampicillin and penicillin G; enrofloxacin (EFX); gentamicin (GEN); sulfa-trimethoprim (SFT); tetracycline (TET); and cephalosporin (CEP). Resistance patterns summarized for cefoxitin, cefotaxime, ceftiofur, ceftriaxone, and cephalexin as well as erythromycin (ERT) and vancomycin (VAN).

Antibiotic Class	MDR Pattern
3 classes	PEN-EFX-GEN (*n* = 2), PEN-GEN-SFT (*n* = 1), PEN-GEN-TET (*n* = 1), PEN-EFX-CEP (*n* = 4), EFX-GEN-CEP (*n* = 2), PEN-TET-CEP (*n* = 3), SFT-TET-CEP (*n* = 2), PEN-CEP-ERT (*n* = 2), GEN-CEP-ERT (*n* = 1), TET-CEP-ERT (*n* = 14), PEN-CEP-VAN (*n* = 1), SFT-CEP-VAN (*n* = 10), SFT-ERT-VAN (*n* = 1), CEP-ERT-VAN (*n* = 4)
4 classes	PEN-EFX-GEN-SFT (*n* = 1), PEN-SFT-TET-CEP (*n* = 3), PEN-GEN-CEP-ERT (*n* = 1), PEN-TET-CEP-ERT (*n* = 1), GEN-SFT-CEP-VAN (*n* = 1), TET-CEP-ERT-VAN (*n* = 2)
5 classes	PEN-EFX-GEN-SFT-TET (*n* = 1), PEN-TET-CEP-ERT-VAN (*n* = 3)

**Table 5:** Prediction of antibiotic resistance for each multidrug-resistant bacterium (MDR).

Antibiotics	Total	Sensitivity	Specificity	PPV	NPV	Accuracy
Penicillin	686	39.3	89.9	27.6	93.8	85.4
Enrofloxacin	205	83.3	89.6	33.3	98.9	89.3
Gentamicin	296	57.9	87.7	24.4	96.8	85.8
Sulfa-trimethoprim	118	90.9	65.4	21.3	98.6	67.8
Tetracycline	442	88.9	69.0	24.5	98.2	71.0
Cephalosporin	686	88.5	84.3	35.5	98.7	84.7
Erythromycin	375	67.4	84.6	36.3	95.3	82.7
Vancomycin	338	56.4	93.3	52.4	94.3	89.1

**Table 6:** The final model analyzed using repeated logistic regression to assess factors associated with vancomycin-resistant bacteria in milk samples from cows on dairy farms in tropical settings (*n* = 338: *n* = 296 susceptible and *n* = 42 resistant).

			Standard		95% Confidence			
Parameter	Level	Estimate	Error	OR	Lower	Upper	Z-Value	*p*-Value
Intercept		−4.2598	0.7335	0.014	0.003	0.059	−5.81	<0.0001
HH farm	No	1.395	0.630	4.035	1.175	13.860	2.22	0.0267
	Yes	Reference						
Year Participated	2020	1.529	0.506	4.611	1.709	12.441	3.02	0.002
	2023	Reference						

**Table 7:** The final model, analyzed using repeated logistic regression, for the factors associated with multidrug-resistant bacteria (MDR) in milk samples from cows on dairy farms in a tropical environment, using data with at least 3 antibiotic classes for the susceptibility test at the same time (*n* = 686: *n* = 625 w/o MDR and *n* = 61 w MDR). The interaction of the HH farm and the participating year was used instead of the separate factors due to its significant effect.

			Standard		95% Confidence			
Parameter	Level	Estimate	Error	OR	Lower	Upper	Z-Value	*p*-Value
Intercept		−1.466	0.199	0.231	0.156	0.341	−7.360	<0.0001
*S* *. uberis*		−1.403	0.641	0.246	0.070	0.863	−2.190	0.029
*S* *. dysgalactiae*		1.027	0.459	2.792	1.136	6.861	2.240	0.025
Participated Year—HH Farm	2023—Yes	−2.001	0.482	0.135	0.053	0.348	−4.150	<0.0001
	2023—No	−1.693	0.541	0.184	0.064	0.531	−3.130	0.002
	2020—Yes	−2.728	0.585	0.065	0.021	0.206	−4.660	<0.0001
	2020—No	Reference						

## 4. Discussion

Pathogen distribution reflects both the microbiological profile of dairy mastitis in central Thailand and differences in diagnostic capacity between farm types. The predominance of *Streptococcus* spp. and CNS is consistent with previously reported profiles in Thai dairy operations [7,9]. The higher prevalence of *S. agalactiae* in HH farm submissions and in the 2023 year group likely reflects more systematic culture practices in program-enrolled farms rather than a true increase in pathogen burden. This is because farms without veterinary oversight rarely perform routine culture before treatment, resulting in the under-detection of contagious pathogens. Similarly, the higher prevalence of *S. uberis* in HH farm submissions (OR = 10.6, *p* < 0.001) reflects culture-based surveillance frequency; this disproportionate representation directly affects aggregate resistance rates in HH farms, as discussed below.

Yeast was exclusively isolated from non-HH farm submissions throughout the five-year period, which is consistent with the fact that unguided empirical antibiotic use is a key predisposing factor for iatrogenic fungal superinfection. Prolonged broad-spectrum therapy disrupts the mammary gland commensal microbiota, creating the opportunity for yeast proliferation, whereas an improper aseptic technique during intramammary infusion can introduce environmental fungi [23,24]. The absence of yeast in HH farm samples suggests that culture-guided antibiotic selection prevents ecological disruption predisposing to mycotic superinfection [17].

Among the organisms identified, *S. uberis* exhibited the broadest descriptive resistance profile. The high tetracycline resistance rate (68.4%) is consistent with Zhang et al. [5], who reported 82.0% in northern Thailand, and Camsing et al. [8], who identified tetracycline resistance as the dominant phenotype among streptococcal mastitis isolates in Sakon Nakhon Province. The elevated resistance to aminoglycoside (gentamicin, 44.4%; OR = 7.6) substantially exceeds rates in European contexts, where gentamicin resistance in *S. uberis* typically remains below 5% [25], suggesting overuse of aminoglycosides in this region. The within-species MDR rate (11.5%) was the highest among species. However, in the adjusted regression model (Table 7), *S. uberis* was associated with lower rather than higher odds of MDR (OR = 0.246; *p* = 0.029). This apparent paradox reflects the structure of the surveillance dataset rather than a true protective property of the organism: *S. uberis* was disproportionately isolated from HH farm submissions in 2023 (OR = 10.6, *p* < 0.01), and constituted the most strongly protective property of the HH × year interaction. After adjusting for this co-occurrence, the residual species-level effect for *S. uberis* shifted to a protective estimate. Therefore, these descriptive findings—broad single-class resistance and the highest within-species MDR rate—remain clinically relevant for empirical-treatment guidance, and the adjusted model identifies HH × year, rather than individual species, as the dominant determinant of the odds of experiencing MDR in this dataset. By contrast, *S. agalactiae* demonstrated uniform susceptibility to various types of penicillin, tetracyclines, and macrolides throughout the surveillance period. This is consistent with the findings of Wataradee et al. [26] in the same region, supporting continued penicillin-based first-line therapy for this pathogen. Resistance to staphylococcal penicillin (CNS 52.8%; *S. aureus* 63.2%) is consistent with the well-documented prevalence of beta-lactamase-producing staphylococci globally [16], underscoring the importance of culture confirmation before initiating penicillin therapy.

The most consistent finding from stratified analysis was the substantial decline in resistance across multiple classes between year groups. Resistance to tetracycline, erythromycin, and vancomycin declined from 47.5% to 13.1%, 26.2% to 10.9%, and 15.9% to 5.4%, respectively. These reductions coincided with the establishment of the TDRC regional center and Dairy School program in 2023, supporting the hypothesis that extending veterinary oversight and enhancing farmer education can reduce antibiotic selection pressure at the population level. The regional tetracycline resistance documented by Camsing et al. [8] in Sakon Nakhon underscores that high tetracycline resistance is a broader challenge across Thai dairy systems, making the observed decline particularly notable. In contrast, enrofloxacin resistance increased from 6.6% to 19.4%. Contributing factors may include the increased proportion of CM submissions in the 2023 year group and enrolment of new farms with histories of empirical fluoroquinolone use prior to program entry; however, the small number of tested isolates (*n* = 206) limits the reliability of this trend. As fluoroquinolones are classified as critically important for human medicine [27], this finding warrants continued surveillance. The principal driver of this resistance is empirical antibiotic use without bacteriological confirmation: smallholder producers routinely initiate therapy based on clinical signs or a positive California Mastitis Test (CMT) alone [19,20,28]. This practice leads to therapeutic failure from inappropriate drug selection and intensified selective pressure favoring resistant strains [18].

The non-susceptibility of vancomycin was detected in 34.4% of MDR isolates, a finding that warrants attention from a One-Health surveillance perspective. The co-occurrence of non-susceptibility in glycopeptides with resistance to penicillin, tetracyclines, cephalosporins, and macrolides—exemplified by the PEN-TET-CEP-ERT-VAN five-class profile identified in three isolates—represents the most extensive combination of resistance detected in this dataset. However, these findings are based solely on phenotypic disk diffusion and should be interpreted as indicative surveillance signals rather than confirmed resistance mechanisms; as such, molecular characterization of *van* gene clusters would be necessary to establish their true public health significance [29,30]. Nonetheless, should phenotypically resistant strains of this profile enter human clinical environments, therapeutic options could be severely constrained, underscoring the value of continued monitoring [6,14]. This concern is contextually supported by Na et al. [14], who identified vancomycin-resistant *S. aureus* in Thai dairy herds, and Wataradee et al. [26], who documented vancomycin-resistant *S. agalactiae* among MDR isolates from central Thai dairy herds. Such studies collectively indicate that CIA-resistant phenotypes have been detected across dairy systems in Thailand. Molecular characterization of determinants of resistance, including *van* gene clusters, remains necessary to identify the true epidemiological and public health significance of these phenotypic findings, both in the present study and in the broader regional literature. From a practical standpoint, the high specificity of vancomycin resistance as an MDR screening indicator (93.3%; PPV 52.4%) suggests that its detection could serve as a useful trigger for comprehensive susceptibility testing in resource-limited diagnostic settings. Phenotypic results alone are insufficient to draw definitive public health conclusions.

The primary findings support the hypothesis that participation in the HH program and year group are independently associated with reduced odds of vancomycin resistance and MDR, an association that, given the observational design, should not be interpreted as direct evidence of a causal protective effect. Non-HH farms were associated with approximately 4-fold higher odds of vancomycin resistance (OR = 4.035; 95% CI: 1.175–13.860), while isolates from early periods had 4.6-fold higher odds of resistance (OR = 4.611; 95% CI: 1.709–12.441). However, the relatively wide confidence intervals reflect the limited number of resistant isolates used (*n* = 42) and warrant cautious interpretation. For MDR, because HH participation and year group interacted significantly, their joint effect was modeled rather than used as separate main effects. Compared with non-HH farms in the early period (reference), the three other groups were associated with significantly lower odds of MDR for this interaction: HH farms in 2023 (OR = 0.135; 95% CI: 0.053–0.348), HH farms in 2020 (OR = 0.065; 95% CI: 0.021–0.206), and non-HH farms in 2023 (OR = 0.184; 95% CI: 0.064–0.531). The HH-2020 groups differed significantly from the reference category; however, their wider confidence interval (0.021–0.206) reflects a smaller sample size and warrants cautious interpretation. The observational design of this study means that these patterns cannot be attributed to the HH program alone. The coincidence of the establishment of the TDRC regional center and Dairy School in 2023 precludes attributing these results to any single intervention. The observed associations are consistent with evidence that culture-guided selective treatment reduces intramammary antibiotic use by >50% without compromising outcomes [17], as well as documented associations between empirical use and AMR amplification in Thai dairy systems [19,20,28]. To our knowledge, this represents the first quantitative evidence from Thailand that participation in a structured veterinary HH program is independently associated with reduced CIA resistance and MDR in dairy mastitis pathogens. These findings provide preliminary support for considering such programs as part of a broader antimicrobial stewardship strategy in tropical smallholder systems [18], though prospective studies with standardized designs will be necessary to establish whether this association reflects a reproducible program effect.

Two findings warrant contextual interpretation. First, in the adjusted model, *S. dysgalactiae* was associated with higher odds of MDR (OR = 2.792; *p* = 0.025), despite its relatively narrow resistance profile dominated by tetracycline resistance (40.6%). This inversion reflects confounding by year group: because *S. dysgalactiae* declined sharply in the 2023 period (OR = 0.3) and was therefore concentrated in the early period—which carried the highest background MDR odds—the adjusted model partitions its temporal co-distribution away from the year-group term, causing the residual species-level estimate to shift upward. Conversely, *S. uberis* was associated with lower adjusted odds of MDR (OR = 0.246; *p* = 0.029) despite carrying the highest within-species MDR rate (11.5%). This reflects its disproportionate concentration in HH-2023 submissions (OR = 10.6, *p* < 0.01): once the HH × year interaction term absorbs the strong protective effect of that group, the residual *S. uberis* estimate is attenuated downward. These adjusted estimates therefore reflect each species’ relative contribution after controlling for the HH × year interaction, rather than the absolute burden of resistance—a distinction that should be retained when translating findings to clinical decisions. Second, the paradoxically higher tetracycline resistance in HH farm isolates at the bivariate level is explained by the same overrepresentation of *S. uberis* in HH submissions; given this species’ tetracycline resistance rate of 68.4%, its concentration in this group elevates aggregate resistance, masking a stewardship effect that is nonetheless evident in the significant reduction in erythromycin resistance among HH farm isolates.

Several limitations of the study design should be acknowledged. This study relied on consecutive diagnostic submissions from a single veterinary program, representing a convenience sample; findings may therefore not generalize to farms without prior veterinary engagement or to other regions. Participation in the herd health program was voluntary, and farms that chose to enroll may have differed systematically from non-enrolled farms in baseline management quality, operator motivation, and pre-existing antibiotic use practices—a selection bias that cannot be excluded and may independently account for part of the observed differences in resistance rates, irrespective of program activities. Potential confounders, including herd size, milk yield, dry cow therapy practices, and seasonal variation, were unavailable for inclusion in the regression models. Collectively, these design characteristics mean that the observed associations should not be interpreted as evidence of a causal effect of the program on resistance outcomes.

Several analytical and laboratory limitations also apply. Antimicrobial susceptibility was determined exclusively by phenotypic disk diffusion, precluding characterization of resistance mechanisms; in particular, phenotypic detection of vancomycin non-susceptibility without molecular confirmation of *van* gene clusters limits the interpretation of its public health significance [14,16]. Because antimicrobial agents were selected per isolate by the attending veterinarian (3–5 agents per isolate), the panel tested varied considerably across submissions, and agent-level resistance rates in Table 3 reflect variable denominators that should be compared across groups only with reference to the sample sizes shown. Isolates tested against fewer than three antibiotic classes were excluded from MDR classification, potentially affecting MDR prevalence estimates. Within the HH × year interaction model, sample sizes in some cells—notably the HH-2020 group—were limited, producing wider confidence intervals; these estimates should therefore be interpreted as reflecting the composition of the dataset rather than as precise measures of the program’s effect. Future studies should employ whole-genome sequencing to characterize resistance gene carriage and dissemination, apply a standardized minimum AST panel, and incorporate economic analyses to quantify the cost-effectiveness of program-associated stewardship outcomes.

## 5. Conclusions

In this five-year surveillance study, participation in a structured veterinary herd health program was independently associated with lower odds of phenotypic vancomycin resistance (non-HH farms: OR = 4.035; 95% CI: 1.175–13.860; *p* = 0.027). The interaction between program participation and surveillance period was statistically significant for MDR; compared with non-HH farms in the early period, all other HH-year combinations were associated with significantly lower odds of MDR (OR range: 0.065–0.184). *Streptococcus uberis* exhibited the highest within-species descriptive MDR rate (11.5%) and the broadest multi-class resistance phenotype. Yeast was isolated exclusively from non-HH farms. Given disk diffusion limitations for glycopeptides, vancomycin non-susceptibility findings require MIC-based and molecular validation before public health significance can be inferred.

## Data Availability

The data that support the findings of this study are available from the corresponding author upon reasonable request.

## Abbreviations

The following abbreviations are used in this manuscript:

AMR	antimicrobial resistance
AST	antimicrobial susceptibility testing
CAMP	Christie–Atkins–Munch-Petersen
CEP	cephalosporin
CIA	critically important antimicrobial
CLSI	Clinical and Laboratory Standards Institute
CM	clinical mastitis
CMT	California mastitis test
CNS	coagulase-negative staphylococci
CU	Chulalongkorn University
EFX	enrofloxacin
ERT	erythromycin
GEN	gentamicin
MDR	multidrug-resistant
MRSA	methicillin-resistant *Staphylococcus aureus*
NMC	National Mastitis Council
NPV	negative predictive value
PEN	penicillin
PPV	positive predictive value
RDU	rational drug use
SCC	somatic cell count
SCM	subclinical mastitis
SFT	sulfa-trimethoprim
TET	tetracycline
TDRC	Dairy Research and Technology Transfer for Tropical Dairy Development Center
VAN	vancomycin
VRE	vancomycin-resistant enterococci

## References

[1] Hogeveen H., Huijps K., Lam T. (2011). Economic aspects of mastitis: New developments. N. Z. Vet. J..

[2] Gussmann M., Steeneveld W., Kirkeby C., Hogeveen H., Nielen M., Farre M., Halasa T. (2019). Economic and epidemiological impact of different intervention strategies for clinical contagious mastitis. J. Dairy Sci..

[3] Sharun K., Dhama K., Tiwari R., Gugjoo M.B., Iqbal Yatoo M., Patel S.K., Pathak M., Karthik K., Khurana S.K., Singh R. (2021). Advances in therapeutic and managemental approaches of bovine mastitis: A comprehensive review. Vet. Q..

[4] Ruegg P.L. (2017). A 100-Year Review: Mastitis detection, management, and prevention. J. Dairy Sci..

[5] Zhang T., Niu G., Boonyayatra S., Pichpol D. (2021). Antimicrobial resistance profiles and genes in *Streptococcus uberis* associated with bovine mastitis in Thailand. Front. Vet. Sci..

[6] Boonyayatra S., Wongsathein D., Tharavichitkul P. (2020). Genetic relatedness among *Streptococcus agalactiae* isolated from Cattle, Fish, and Humans. Foodborne Pathog. Dis..

[7] Boonyayatra S., Pata P., Nakharuthai P., Chaisri W. (2016). Antimicrobial resistance of biofilm-forming *Streptococcus agalactiae* isolated from bovine mastitis. J. Vet. Sci. Technol..

[8] Camsing A., Phetburom N., Chopjitt P., Pumhirunroj B., Patikae P., Watwiengkam N., Yongkiettrakul S., Kerdsin A., Boueroy P. (2024). Occurrence of antimicrobial-resistant bovine mastitis bacteria in Sakon Nakhon, Thailand. Vet. World.

[9] Wataradee S., Samngamnim S., Boonserm T., Ajariyakhajorn K. (2023). Genotypic and antimicrobial susceptibility of *Streptococcus agalactiae* causing bovine mastitis in the central region of Thailand. Front. Vet. Sci..

[10] Salam M.A., Al-Amin M.Y., Salam M.T., Pawar J.S., Akhter N., Rabaan A.A., Alqumber M.A.A. (2023). Antimicrobial Resistance: A Growing Serious Threat for Global Public Health. Healthcare.

[11] Tenover F.C., Biddle J.W., Lancaster M.V. (2001). Increasing resistance to vancomycin and other glycopeptides in *Staphylococcus aureus*. Emerg. Infect. Dis..

[12] Velazquez-Meza M.E., Galarde-López M., Carrillo-Quiróz B., Alpuche-Aranda C.M. (2022). Antimicrobial resistance: One health approach. Vet. World.

[13] Park C., Nichols M., Schrag S.J. (2014). Two cases of invasive vancomycin-resistant group B streptococcus infection. N. Engl. J. Med..

[14] Na S., Intanon M., Srithanasuwan A., Chaisri W., Suriyasathaporn W. (2025). Evidence of vancomycin-resistant *Staphylococcus aureus*, multidrug-resistant *S. aureus*, and *Enterococcus faecium*-causing mastitis in Thailand and Cambodia. Vet. World.

[15] Magiorakos A.-P., Srinivasan A., Carey R.B., Carmeli Y., Falagas M., Giske C., Harbarth S., Hindler J., Kahlmeter G., Olsson-Liljequist B. (2012). Multidrug-resistant, extensively drug-resistant and pandrug-resistant bacteria: An international expert proposal for interim standard definitions for acquired resistance. Clin. Microbiol. Infect..

[16] Barlow J. (2025). Antimicrobial Resistance of Mastitis Pathogens of Dairy Cattle. Vet. Clin. Food Anim. Pract..

[17] Lago A., Godden S.M. (2018). Use of rapid culture systems to guide clinical mastitis treatment decisions. Vet. Clin. Food Anim. Pract..

[18] Cole J., Mughal A.N., Eltholth M., Thomas A., Holmes M. (2024). Transdisciplinary approaches to addressing factors that influence antimicrobial use in dairy cattle: A scoping review. Heliyon.

[19] Ratanapob N., Saengtienchai A., Rukkwamsuk T. (2024). Knowledge, Attitude, and Practice of Thai Dairy Farmers on the Use of Antibiotics. Vet. Med. Int..

[20] Dankar I., Hassan H., Serhan M. (2022). Knowledge, attitudes, and perceptions of dairy farmers regarding antibiotic use: Lessons from a developing country. J. Dairy Sci..

[21] Adkins P.R., Middleton J.R. (2017). Laboratory Handbook on Bovine Mastitis.

[22] CLSI (2018). Performance Standards for Antimicrobial Disk and Dilution Susceptibility Tests for Bacteria Isolated from Animals.

[23] Scaccabarozzi L., Locatelli C., Pisoni G., Manarolla G., Casula A., Bronzo V., Moroni P. (2011). Epidemiology and genotyping of Candida rugosa strains responsible for persistent intramammary infections in dairy cows. J. Dairy Sci..

[24] Gaudie C., Wragg P., Barber A. (2009). Outbreak of disease due to Candida krusei in a small dairy herd in the UK. Vet. Rec..

[25] Miotti C., Cicotello J., Archilla G.S., Neder V., Lucero W.A., Calvinho L., Signorini M., Camussone C., Zbrun M.V., Molineri A.I. (2023). Antimicrobial resistance of *Streptococcus uberis* isolated from bovine mastitis: Systematic review and meta-analysis. Res. Vet. Sci..

[26] Wataradee S., Boonserm T., Samngamnim S., Ajariyakhajorn K. (2024). Characterization of virulence factors and antimicrobial susceptibility of streptococcus agalactiae associated with bovine mastitis cases in Thailand. Animals.

[27] World Health Organization (2019). WHO List of Critically Important Antimicrobials for Human Medicine (WHO CIA List).

[28] Haenssgen M.J., Charoenboon N., Zanello G., Mayxay M., Reed-Tsochas F., Lubell Y., Wertheim H., Lienert J., Xayavong T., Zaw Y.K. (2019). Antibiotic knowledge, attitudes and practices: New insights from cross-sectional rural health behaviour surveys in low-income and middle-income South-East Asia. BMJ Open.

[29] Kest H., Kaushik A. (2019). Vancomycin-resistant Staphylococcus aureus: Formidable threat or silence before the storm. J. Infect. Dis. Epidemiol..

[30] Rengaraj R., Mariappan S., Sekar U., Kamalanadhan A. (2016). Detection of vancomycin resistance among *Enterococcus faecalis* and *Staphylococcus aureus*. J. Clin. Diagn. Res..

